# Horizontal Transmission of the Symbiont *Microsporidia MB* in *Anopheles arabiensis*

**DOI:** 10.3389/fmicb.2021.647183

**Published:** 2021-07-28

**Authors:** Godfrey Nattoh, Tracy Maina, Edward E. Makhulu, Lilian Mbaisi, Enock Mararo, Fidel G. Otieno, Tullu Bukhari, Thomas O. Onchuru, Evan Teal, Juan Paredes, Joel L. Bargul, David M. Mburu, Everline A. Onyango, Gabriel Magoma, Steven P. Sinkins, Jeremy K. Herren

**Affiliations:** ^1^International Centre of Insect Physiology and Ecology (icipe), Nairobi, Kenya; ^2^Institute for Basic Sciences Technology and Innovation, Pan African University, Nairobi, Kenya; ^3^Research Unit in Bioinformatics (RUBi), Department of Biochemistry and Microbiology, Rhodes University, Grahamstown, South Africa; ^4^The Royal (Dick) School of Veterinary Studies, Roslin Institute, The University of Edinburgh, Edinburgh, United Kingdom; ^5^Department of Physical and Biological Sciences, Bomet University College, Bomet, Kenya; ^6^Pwani University Biosciences Research Centre (PUBReC), Kilifi, Kenya; ^7^Kemri-Wellcome Trust Research Program, Kilifi, Kenya; ^8^Department of Biochemistry, Jomo Kenyatta University of Agriculture and Technology, Nairobi, Kenya; ^9^MRC-University of Glasgow Centre for Virus Research, Glasgow, United Kingdom

**Keywords:** symbiosis, *Anopheles*, malaria, vector, *Microsporidia*

## Abstract

The recently discovered *Anopheles* symbiont, *Microsporidia MB*, has a strong malaria transmission-blocking phenotype in *Anopheles arabiensis*, the predominant *Anopheles gambiae* species complex member in many active transmission areas in eastern Africa. The ability of *Microsporidia MB* to block *Plasmodium* transmission together with vertical transmission and avirulence makes it a candidate for the development of a symbiont-based malaria transmission blocking strategy. We investigate the characteristics and efficiencies of *Microsporidia MB* transmission between *An. arabiensis* mosquitoes. We show that *Microsporidia MB* is not transmitted between larvae but is effectively transmitted horizontally between adult mosquitoes. Notably, *Microsporidia MB* was only found to be transmitted between male and female *An. arabiensis*, suggesting sexual horizontal transmission. In addition, *Microsporidia MB* cells were observed infecting the *An. arabiensis* ejaculatory duct. Female *An. arabiensis* that acquire *Microsporidia MB* horizontally are able to transmit the symbiont vertically to their offspring. We also investigate the possibility that *Microsporidia MB* can infect alternate hosts that live in the same habitats as their *An. arabiensis* hosts, but find no other non-anopheline hosts. Notably, *Microsporidia MB* infections were found in another primary malaria African vector, *Anopheles funestus s.s*. The finding that *Microsporidia MB* can be transmitted horizontally is relevant for the development of dissemination strategies to control malaria that are based on the targeted release of *Microsporidia MB* infected *Anopheles* mosquitoes.

## Importance Statement

The malaria disease burden remains a major impediment to good health and economic development in many regions of sub-Saharan Africa. We have recently reported that a microsporidian symbiont (*Microsporidia MB*) naturally blocks *Plasmodium* transmission in *Anopheles arabiensis*, a major vector of malaria in Africa. *Microsporidia MB* could form the basis of a novel transmission blocking intervention for malaria control. However, the development of *Microsporidia MB* as an intervention a strategy will require a better understanding of the symbiont’s biology. Of particular relevance are the natural mosquito to mosquito transmission routes that enable *Microsporidia MB* to spread within *Anopheles* mosquito populations and which could potentially be used to disseminate *Microsporidia MB* as part of a malaria transmission blocking strategy. We investigate the natural routes of *Microsporidia MB’s* mosquito to mosquito transmission and find that it can be transmitted horizontally between adult *An. arabiensis* of opposite sexes. This finding will aid the development of a *Microsporidia MB* dissemination strategy, potentially involving targeted release of *Microsporidia MB* infected *Anopheles* mosquitoes.

## Introduction

Malaria continues to be a major health threat across sub-Saharan Africa, with this region accounting for 93% of the global malaria deaths (World Health Organization, 2020). The major preventive strategies for malaria control remain the use of long-lasting insecticidal nets (LLINs) and indoor residual spraying (IRS). In conjunction with improvements in case detection and management, these strategies have reduced malaria cases by up to 40% between 2000 and 2015 (Bhatt et al., 2015). However, progress has plateaued and possibly reversed, with case levels remaining the same between 2014 and 2016 and increasing between 2016 and 2017 (D’Alessandro, 2018; World Health Organization, 2020). It is apparent that current malaria control strategies have their limitations and there is a vital need for complementary tools (Huijben and Paaijmans, 2018).

The malaria transmission cycle relies on female *Anopheles* mosquitoes becoming infected by feeding on human blood that contains the *Plasmodium* gametocyte stage. *Plasmodium* gametocytes undergo a series of developmental changes before traversing the mosquito midgut to form a sporogonic oocyst, which produces sporozoites that are released into the mosquito hemocoel. Sporozoites in the hemocoel travel to the mosquito salivary glands to enter the mosquito’s saliva, which results in an infected mosquito, usually 8–14 days after the bloodmeal (Baton and Ranford-Cartwright, 2005). This transmission cycle can be impeded by inhibitory interactions with mosquito-associated microbes (Romoli and Gendrin, 2018). One of the most promising new management strategies involves the use of vertically (mother to offspring) transmitted symbiotic microbes that prevent the establishment of disease-causing viruses in mosquito vectors. This strategy is currently used as a control mechanism against the arboviral disease, Dengue, through the bacterial symbiont, *Wolbachia* (Moreira et al., 2009; Bian et al., 2010; Hoffmann et al., 2011; Walker et al., 2011; Frentiu et al., 2014; Ant et al., 2018; Nazni et al., 2019).

The *Anopheles*-associated symbiont *Microsporidia MB* colonizes mosquito ovaries and is vertically transmitted. This microsporidian can also block the transmission of malaria by *Anopheles* mosquitoes (Herren et al., 2020), and therefore could potentially contribute to the control of malaria. The successful deployment of symbiont-based vector-borne disease control strategies requires the ability to spread symbionts through host insect populations and the maintenance of a high prevalence of infection. In *Wolbachia-*based strategies, cytoplasmic incompatibility can effectively drive symbionts through mosquito populations. In the absence of cytoplasmic incompatibility, other driving mechanisms would be required to spread *Microsporidia MB* through *Anopheles* populations. *Microsporidia MB* is naturally found in populations of *Anopheles* mosquitoes in Kenya, ranging in prevalence from 0 to 25% (Herren et al., 2020). From the standpoint of symbiont-based control strategies, the different *Microsporidia MB* transmission routes could be relevant for interventions that could generate a higher prevalence of the transmission-blocking symbiont in *Anopheles* mosquito populations, leading to reductions in malaria transmission.

Microsporidia are a diverse clade of obligate, intracellular organisms that infect an array of hosts, including vertebrates and invertebrates and are found in both terrestrial and aquatic environments (Vossbrinck and Debrunner-Vossbrinck, 2005). The morphology of Microsporidia can be simplified into the meront phase, which is present during proliferation, and the spore, which is resistant to environmental degradation and transmission-specialized. Microsporidian spores are characterized by a chitinous wall and a polar filament involved in host cell penetration (Stentiford et al., 2013). In arthropods, Microsporidian transmission can occur vertically (mother to offspring) and horizontally (from one individual to another of the same generation, Stentiford et al., 2013). There are also many reported incidences of microsporidians using a combination of vertical and horizontal transmission. Vertical transmission generally occurs via the transovarial route with spores germinating on the periphery or inside of ovaries to colonize developing eggs. Vertical transmission is associated with greater host specificity and lower Microsporidia burden and virulence (Vávra and Lukeš, 2013). There are different forms of horizontal transmission in arthropod-associated Microsporidia, however the most widespread is oral and involves the ingestion of spores, which subsequently germinate and inject their sporoplasm into the host intestinal cells through a polar filament. Microsporidia that predominately rely on oral horizontal transmission tend to be associated with lower levels of host specificity and high virulence as microsporidian spores will usually be released en masse from deceased hosts to infect other hosts (Han and Weiss, 2017). Other forms of horizontal transmission that are not associated with high virulence, for example sexual transmission, have also been demonstrated in several microsporidian species. *Nosema plodiae* is a microsporidian pathogen of the Indian meal moth, *Plodia interpunctella*, which invades the reproductive organs of its host and is transmitted from male to female moths during mating (Kellen and Lindegren, 1971).

The Microsporidia transmission mode influences host specificity and life-cycle complexity (Stentiford et al., 2013). Microsporidians can be generalists, infecting a variety of different hosts or exhibit high levels of host specialization. Microsporidians can have specialization toward a single (simple lifecycle) or several intermediate hosts (complex lifecycle). Vertical and sexual transmission result in limited opportunities for Microsporidia to infect hosts of a different species and are therefore likely to lead to higher levels of host specificity. In contrast, horizontal transmission by spore ingestion is likely to be associated with lower levels of host specificity. Microsporidians with simple and complex lifecycles can use both vertical and horizontal transmission. In most cases, different spores types become specialized for different transmission routes (Stentiford et al., 2013).

We investigated a number of possible horizontal transmission routes for *Microsporidia MB* in *An. arabiensis*. We established that transmission was only found to occur between adult mosquitoes. In addition, transmission was only observed between different sexes, which indicates that *Microsporidia MB* is sexually transmitted in *An. arabiensis*.

## Results

### Horizontal Transmission of *Microsporidia MB* Occurs Between Adult *An. arabiensis*

**FIGURE 1:**
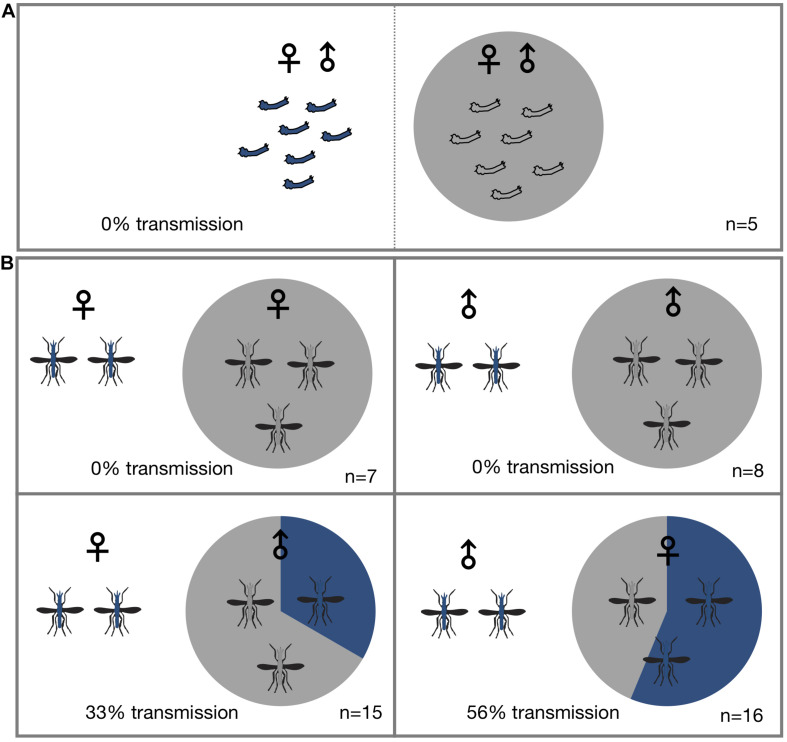
Horizontal transmission of *Microsporidia MB.* Mosquitoes carrying *Microsporidia MB* are represented with blue shading in pie charts and n = number of independent experiments. **(A)** No transmission of *Microsporidia MB* was observed between *An. arabiensis* larvae reared in the same larval trough but separated by a screen mesh. **(B)** Horizontal transmission of *Microsporidia MB* was observed when adults were kept together in cages, and specifically when either infected males or females were housed with uninfected *An. arabiensis* of the opposite sex. Top row, no transmission was observed between infected and uninfected individuals of the same sex. Bottom left, transmission between *Microsporidia MB* infected *An. arabiensis* females and uninfected males was observed in 5 out of 15 cages (33%). Bottom right, out of a total of 16 experiments that had *Microsporidia MB* infected males and uninfected females and horizontal transmission was confirmed in 10 of these cages (56% transmission).

To determine if *Microsporidia MB* is horizontally transmitted at the adult or larval stages, *Microsporidia MB* infected and uninfected larvae and mosquitoes were housed together in larval rearing troughs or cages. Since it is difficult to reliably mark or determine the sex of larvae, we placed infected and uninfected larvae in two adjacent sections of rearing trough that was separated by a screen mesh. For larval experiments a roughly equal number of infected donor and uninfected recipient L1 larvae (*N* = 16–35) were placed in mesh separated compartments and allowed to develop into adults. After adults eclosed both donor and recipient specimens were screened for the presence of *Microsporidia MB*. Under these conditions horizontal transmission of *Microsporidia MB* was not observed (Figure 1A and Table 1). The addition of homogenized infected larvae to the rearing water of uninfected larvae and to sugar sources given to uninfected adult *An. arabiensis* also did not result in horizontal transmission of *Microsporidia MB* (Table 2). Altogether these findings indicate that intact, alive *An. arabiensis* larvae or the homogenates of *Microsporidia MB*-infected larvae and adults are not able to transmit *Microsporidia MB* horizontally to other *An. arabiensis* individuals (larval or adult).

**TABLE 1:** Horizontal transmission is not observed when *An. arabiensis* larvae are reared in the same larval trough but separated by a screen mesh.

Expt #	Number of	Microsporidia MB positives	Number of	Infection prevalence	Transmission
(Sheet labels)	donor larvae	in donor larvae	recipient larvae	in donor larvae	rate
LL1	10	8	14	0	0
LL3	9	9	20	0	0
LL4	14	4	31	0	0
LL5	20	13	10	0	0
LL6	7	7	9	0	0

**TABLE 2:** Homogenates from larval and adult *Microsporidia MB* infected mosquitoes are not able to establish infections after being ingested by *An. arabiensis*.

Source of *Microsporidia MB* inoculum	Target *Anopheles* stage	Number of experimental repeats	Number of samples per experiment	*Microsporidia MB* Transmission
Homogenized larvae	Larvae (in rearing water)	3	20,14,17	0/20, 0/14, and 0/17
Homogenized larvae	Adults (in sugar source)	4	13,16,16	0/13, 0/16, and 0/16
Homogenized adults	Adults (in sugar source)	3	31,36,40	0/31, 0/36, and 0/40
Homogenized adults	Larvae (in rearing water)	3	28,31,28	0/28, 0/31, and 0/28

*Homogenized infected larvae and adults were fed to adult (in sugar source) and larval (in rearing water) *An. arabiensis*, to determine if *Microsporidia MB* could be transmitted horizontally by ingestion. None of the *An. arabiensis* that fed on *Microsporidia MB* infected homogenates became infected with *Microsporidia MB.**

To investigate horizontal transmission of *Microsporidia MB* between live adults, we established cages with *Microsporidia MB* infected and uninfected mosquitoes. Adult mosquitoes were maintained in these cages for a period of 2 days before they were screened for the presence of *Microsporidia MB*. Additionally, to determine if horizontal transmission between mosquitoes could involve sugar sources, these were screened; *Microsporidia MB* was not detected in sugar sources (Table 3). In cages that had *Microsporidia MB* infected and uninfected mosquitoes of the same sex, the mosquitoes were marked with dye to indicate *Microsporidia MB* “donors” and “recipients” prior to exposure. In general, 2–6 infected *An. arabiensis* were kept together with 10–25 uninfected mosquitoes in standard 30 cm × 30 cm × 30 cm cages. At the end of the experiment all mosquitoes were screened to confirm infection status and determine if horizontal transmission had occurred. Out of 47 cage experiments, horizontal transmission was observed in 15 cage experiments (Figure 1B and Table 4). Notably, horizontal transmission was only observed in cages that had opposite sexes of *Microsporidia MB* infected and uninfected adult *An. arabiensis*. Out of 16 cages that had *Microsporidia MB* infected males and uninfected females, transmission was confirmed in 9 cages (56%). Amongst 15 cages that had *Microsporidia MB* infected females and uninfected males, transmission was confirmed in 5 cages (33%). In 15 cages that had the same sex *Microsporidia MB* infected and uninfected adult *An. arabiensis*, horizontal transmission was not observed. To investigate the link between *Microsporidia MB* transmission from male *An. arabiensis* to females and insemination, and to approximate the mating frequency in cage experiments, female *An. arabiensis* spermatheca were dissected and checked for the presence of sperm (Table 5). The mating frequency in cage experiments where spermatheca were checked (*N* = 3) was found to range from 0 to 8%. Notably, *Microsporidia MB* transmission was only recorded in females that had sperm in their spermatheca.

**TABLE 3:** Sugar sources fed on by *Microsporidia MB* infected mosquitoes do not contain detectable levels of *Microsporidia MB*.

Experiment ID	Experiment type	Infection status
MF4	Male + /Female −	Not infected
MF5	Male + /Female −	Not infected
MF6	Male + /Female −	Not infected
MF7	Male + /Female −	Not infected
MF8	Male + /Female −	Not infected
MF9	Male + /Female −	Not infected
MF10	Male + /Female −	Not infected
FM2	Female + /Male −	Not infected
FM3	Female + /Male −	Not infected
FM4	Female + /Male −	Not infected
MM1	Male + /Male −	Not infected
MM2	Male + /Male −	Not infected
MM3	Male + /Male −	Not infected
MM4	Male + /Male −	Not infected
FF1	Female + /Female −	Not infected
FF2	Female + /Female −	Not infected
FF3	Female + /Female −	Not infected

**TABLE 4:** Horizontal transmission of *Microsporidia MB* between adults housed together in cages.

	Expt # (Sheet labels)	Number of donor mates in the cage	Number of confirmed MB+ donor mates in the cage	Total exposed screened	Donor 1 intensity	Donor 2 intensity	Donor 3 intensity	Donor 4 intensity	Donor 5 intensity	Donor 6 intensity	# Recipients acquired Infection	# Recipients didn’t acquire Infection	Recipient 1 intensity	Recipient 2 Intensity	Recipient 3 Intensity
**Male to Female**															
	MF4	2	1	14	6.468956	NA	NA	NA	NA	NA	1	13	15.67		
	MF5	3	1	14	3.950098	NA	NA	NA	NA	NA	1	13	0.659		
	MF6	2	2	14	1.308167	8.4389	2.8418	NA	NA	NA	0	14			
	MF7	2	2	15	12.48482	41.056	6.1403	NA	NA	NA	3	12	2.041	0.8229	0.496
	MF8	2	2	19	4.897402	94.392	8.5958	NA	NA	NA	3	16	0.624	36.948	0.254
	MF9	2	1	14	14.75445	NA	NA	NA	NA	NA	1	13	173.6		
	MF10	2	1	21	0.489231	NA	NA	NA	NA	NA	2	19	14.54	6.3849	
	MF11	2	1	25	1.320645	NA	NA	NA	NA	NA	1	24	4.996		
	MF16	2	1	17	1.329321	NA	NA	NA	NA	NA	0	17			
	MF18	2	1	17	0.224129	NA	NA	NA	NA	NA	0	17			
	MF24	2	1	25	0.148271	NA	NA	NA	NA	NA	0	25			
	MF3	3	1	13	0.476983	NA	NA	NA	NA	NA	0	13			
	SPM 212	7	3	33	2990.757	3.4633	449.23	7.5334	207.08	NA	1	32	354.4		
	SPM 297	7	1	31	11.42972	NA	NA	NA	NA	NA	0	31			
	SPM 304	11	3	37	27.86255	1.1878	16.141	1E-05	3660	NA	2	35	5.132	25.646	
	SPM 315	3	2	17	4.047635	5.3183	2.1773	N/A	N/A	NA	0	17			
**Female to Male**															
	FM2	6	2	20	20.15738	1.6845	NA	NA	NA	NA	0	20			
	FM3	5	1	25	5.352259	NA	NA	NA	NA	NA	0	25			
	FM4	4	1	25	1.467548	NA	NA	NA	NA	NA	0	30			
	BDF03	11	1	16	13.74596	NA	NA	NA	NA	NA	0	16			
	BDF47	13	1	14	7.607281	NA	NA	NA	NA	NA	1	13	0.337		
	BDF53	15	5	13	0.45705	42.397	1.3176	4.1668	44.417	NA	3	10	0.397	1.8002	4.485
	BDF64	9	2	19	2.01744	0.2731	NA	NA	NA	NA	2	17	9.998	1.1225	
	BDF 77	13	2	21	10.08619	0.1461	NA	NA	NA	NA	0	21			
	318 B	15	6	26	25.97944	14.504	9.4598	5.8088	2.2449	7.2807	0	26			
	319A	10	1	43	173.9875	NA	NA	NA	NA	NA	0	43			
	338D	11	4	39	2.583147	4.761	2.2513	1.1604	NA	NA	0	39			
	339A	5	2	19	1305.295	2.6964	NA	NA	NA	NA	1	18	20.43		
	BDF345	4	1	23	7.598649	N/A	NA	NA	NA	NA	0	22			
	BDF346	4	3	11	0.249339	11.79	6.4213	NA	NA	NA	1	10	2.269		
	BDF349	7	6	12	1.095105	1.2174	7.1288	27.826	50.479	1.2548	0	12			
**Male to Male**															
	MM1	2	1	17	0.99955	NA	NA	NA	NA	NA	0	17			
	MM2	3	1	12	2.348149	NA	NA	NA	NA	NA	0	12			
	MM3	1	1	15	6.259642	NA	NA	NA	NA	NA	0	15			
	MM4	2	1	20	3.925768	NA	NA	NA	NA	NA	0	20			
	PPM02	2	1	24	2.349105	NA	NA	NA	NA	NA	0	24			
	PPM08	3	2	31	0.516775	11.651	NA	NA	NA	NA	0	31			
	PPM25	9	1	28	11.96361	NA	NA	NA	NA	NA	0	28			
	PPM31	13	1	13	9.222971	NA	NA	NA	NA	NA	0	13			
**Female to Female**															
	FF1	2	1	13	1.62354	NA	NA	NA	NA	NA	0	13			
	FF2	3	1	25	0.21161	NA	NA	NA	NA	NA	0	25			
	FF3	5	2	16	30.35195	14.257	NA	NA	NA	NA	0	16			
	PPF01	6	5	19	26.56585	21.535	57.306	302.93	51.256	NA	0	19			
	PPF07	4	1	21	1.339635	NA	NA	NA	NA	NA	0	21			
	PPF22	5	1	23	2.30285	NA	NA	NA	NA	NA	0	23			
	PPF32	7	3	19	0.257489	11.032	54.376	NA	NA	NA	0	19			

*Horizontal transmission of *Microsporidia MB* was observed when either infected males or females were housed with uninfected *An. arabiensis* of the opposite sex.*

**TABLE 5:** *Microsporidia MB* transmission is linked to the presence of sperm in female *An. arabiensis* spermatheca.

	# of male donors	# of male donors MB+	# of female recipients	# of female recipients with sperm in spermatheca	# of female recipients sperm+ and MB+	# of female recipients sperm– and MB+
SPC12	3	1	25	2	1	0
SPC09	1	1	40	0	0	0
SPC15	3	2	59	2	1	0

### The Success of *Microsporidia MB* Horizontal Transmission Is Not Linked to Male Infection Intensity

To investigate the factors that influence the rate of *Microsporidia MB* male to female transmission in *An. arabiensis*, we established cages with a single *Microsporidia MB* infected male and 11–48 *Microsporidia MB* uninfected females. Adult mosquitoes were maintained in these cages for a period of 2 days prior to being screened for the presence and intensity of *Microsporidia MB* by quantitative PCR. Out of a total of 33 individual *An. arabiensis* males, 17 were able to infect at least one *An. arabiensis* female in their cage (51.5%). The highest number of females infected by a single male was 3 females. There was no significant link between male intensity of *Microsporidia MB* infection and odds of successfully infecting of one or more uninfected females [exp(b) = 0.982, *P* = 0.715 df = 31]. The number of females per cage did not affect the odds of *Microsporidia MB* transmission to one or more females [exp(b) = 0.968, *P* = 0.421 df = 31]. The correlation between the *Microsporidia MB* infection intensity in donor males and in the female “recipients” was not significantly correlated (*R*^2^ = 0, *P* = 0.34 df = 31) and Supplementary Figure 1). Notably, the average infection intensity in recipient females (10.33) was twice as high as the average infection intensity in male “donors” (5.01).

### *Microsporidia MB* Is Localized to Male *An. arabiensis* Midgut, Gonads, and Seminal Fluid

**FIGURE 2:**
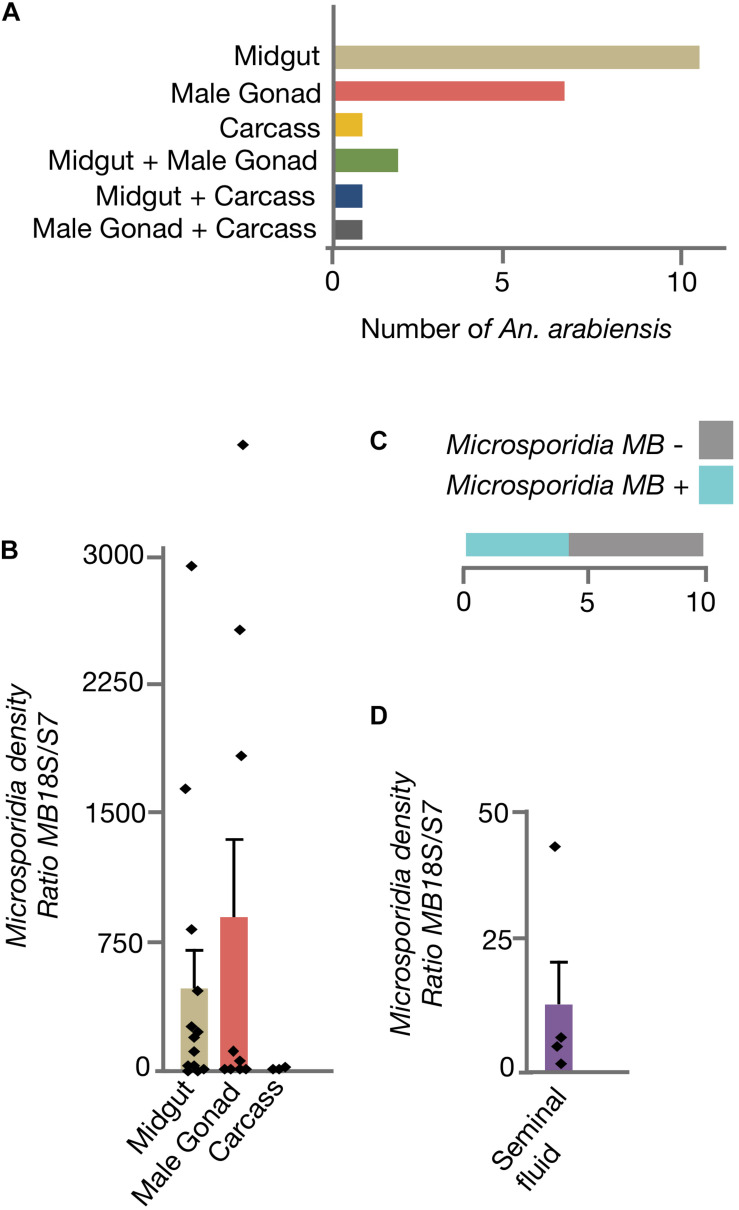
Distribution of *Microsporidia MB* across male *An. arabiensis* organs. **(A)** The screening dissected organs from 22 male *An. arabiensis* specimens, reveals that *Microsporidia MB* is detected primarily in the midguts and male gonads. **(B)** The intensity of *Microsporidia MB* infection is highest in the midgut and male gonads. **(C)** The screening male *An. arabiensis* seminal fluid revealed that *Microsporidia MB* was detected in 4/10 specimens. **(D)** The intensity of *Microsporidia MB* infection in *An. arabiensis* seminal fluid ranges from a ratio of 0.87 to 41.8 MB18S/S7. Error bars reflect SEM.

To determine if *Microsporidia MB* organ distribution in *An. arabiensis* could be linked to transmission routes, adult males were dissected and *Microsporidia MB* intensity was quantified in the midgut, male gonads and carcass (Figure 2A). In the majority of male *An. arabiensis* specimens, *Microsporidia MB* was detected in the midgut (11/22) or male gonads (7/22). In 2/22 specimens, *Microsporidia MB* was detected in both the midgut and the male gonads, whereas in only 3/22 specimens could *Microsporidia MB* be detected in the carcass. In line with these findings, the intensity of *Microsporidia MB* infections were found to be highest in the *An. arabiensis* midgut and male gonads and was found to be lower in carcasses (Figure 2B). The collection of seminal fluid from *Microsporidia MB* infected male *An. arabiensis* revealed that high intensities of *Microsporidia MB* could be detected in seminal fluid collected from 4/10 *An. arabiensis* males (Figures 2C,D).

### *Microsporidia MB* Cells Are Present in the *An. arabiensis* Male Ejaculatory Duct

**FIGURE 3:**
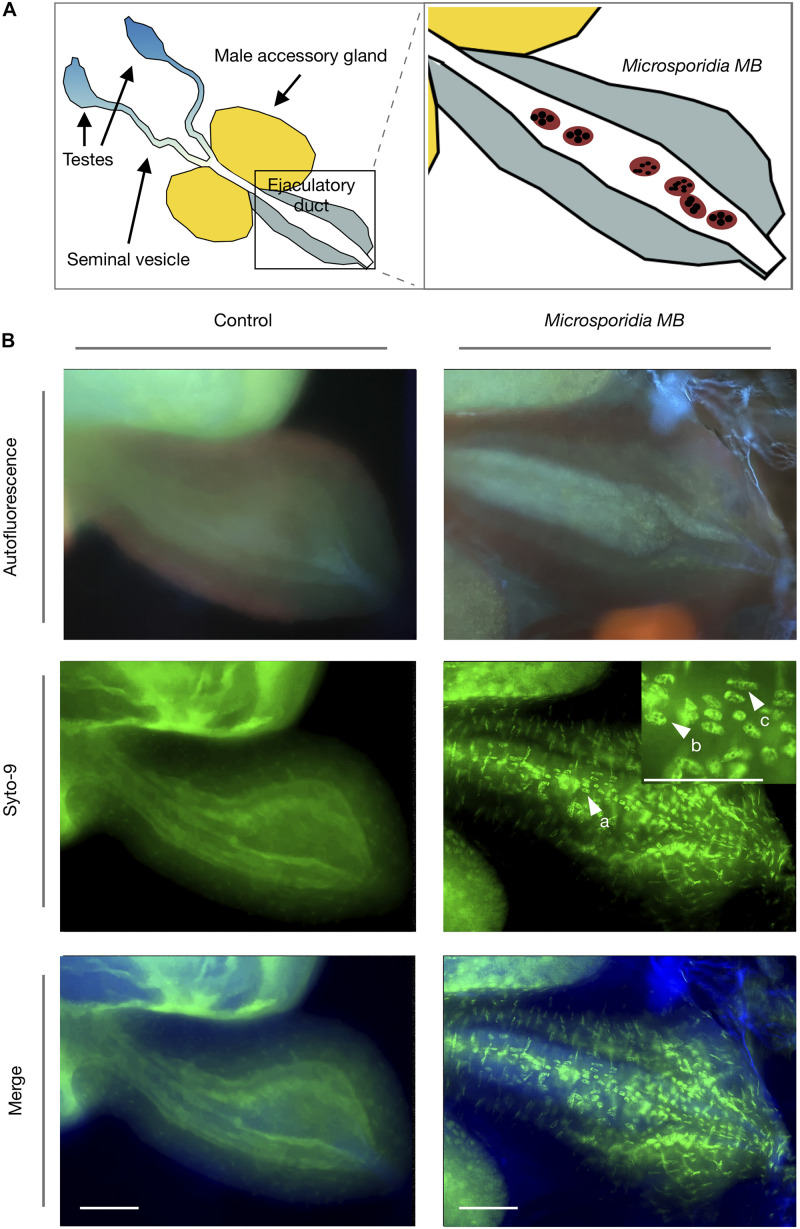
Fluorescence microscopy of *Microsporidia MB* in *An. arabiensis* male ejaculatory ducts. **(A)** Schematic diagram of the male *Anopheles* gonad shows the position of the ejaculatory duct in relation to seminal vesicle, male accessory gland and testes. **(B)** Fluorescence microscopy images indicate that *Microsporidia MB* meronts (a) are found in the male *An. arabiensis* ejaculatory duct. Multinucleate *Microsporidia MB* cells can be observed containing 4 and 8 distinct nuclei (b,c), which likely corresponds to the progression on of 4-nuclei sporogonial plasmodia into the 8-nuclei sporogonial plasmodia and ultimately into sporophorous vesicles. Scale bar = 50 μm.

Fluorescence microscopy of male gonads revealed that *Microsporidia MB* cells were present in the male ejaculatory duct (Figure 3). Only in *Microsporidia MB* infected male *An. arabiensis* were the multinucleated cells corresponding to *Microsporidia MB* observed. Syto-9 nucleic acid staining revealed that the *Microsporidia MB* cells generally had either 4 or 8 nuclei, which likely corresponds to the progression on of 4-nuclei sporogonial plasmodia into an 8-nuclei stage (3rd sporogonic nuclear division) and ultimately becoming sporophorous vesicles (Sokolova and Fuxa, 2008).

### *Microsporidia MB* Can Be Transmitted Vertically After Horizontal Transmission

**FIGURE 4:**
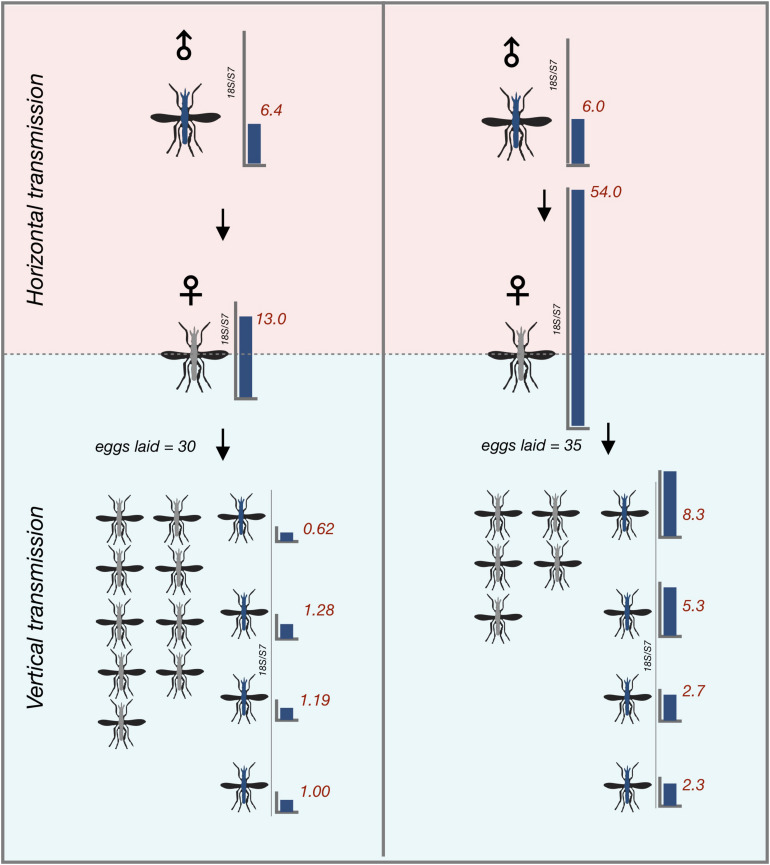
Vertical transmission of horizontally acquired *Microsporidia MB* infections in *An. arabiensis*. Vertical transmission of *Microsporidia MB* is observed in recipient females that have become infected with *Microsporidia MB* after being kept with *Microsporidia MB* infected donor males in the first gonotrophic cycle after horizontal acquisition. Red numbers indicate *Microsporidia MB* infection intensity in individual *An. arabiensis* adults as determined by qPCR.

To determine whether *An. arabiensis* females that horizontally acquired *Microsporidia MB* from the infected males could vertically transfer the infection to their offspring, we gave the recipient *An. arabiensis* females from all single male transmission cages a blood meal and collected eggs from them. Notably, only 4 out of 22 (18%) females successfully acquired a blood meal. Two out of the 4 female *An. arabiensis* that successfully acquired a blood meal laid eggs. Eggs were then allowed to develop into adults prior to being screened. *Microsporidia MB* was detected in 37% of the progeny of recipient female *An. arabiensis* mosquitoes, indicating that *Microsporidia MB* that is horizontally acquired can be subsequently vertically transmitted in the next gonotrophic cycle (Figure 4).

### *Microsporidia MB* Was Not Detected in Potential Secondary Hosts

Since microsporidians can have complex life cycles that involve secondary hosts, we screened a number of other mosquito species and aquatic organisms that inhabit the same habitats as *An. arabiensis* in Western Kenya. *Microsporidia MB* was not detected in mosquitoes in the genus *Aedes* and *Culex* as well as *Culicoides* midges (Table 6). In addition, no *Microsporidia MB* infections were found in crustaceans in the genera *Mesocyclops*, *Macrocyclops* and *Daphnia. Microsporidia MB* was detected in *Anopheles funestus s.s.* but not *Anopheles coustanii*. While this survey of potential secondary hosts was not exhaustive, these findings suggest that *Microsporidia MB* is likely to be an *Anopheles*-specific symbiont.

**TABLE 6:** *Microsporidia MB* was not observed in non-anopheline arthropods from the same habitats as *An. arabiensis*.

Genus of organism	Total number of individuals screened	Collection sites (n)	Presence of *Microsporidia MB*
*Aedes aegypti*	215	Kilifi/Malindi	No infection
*Aedes* sp.	10	Mbita	No infection
*Culex quinquefaciatus*	82	Kilifi/Malindi (15), Nairobi (37), and Mbita (30)	No infection
*Culex* sp.	61	Kilifi/Malindi	No infection
*Culicoides* sp.	42	Ahero (20) and Mwea (22)	No infection
*Anopheles coustanii*	42	Ahero (20) and Mwea (22)	No infection
*Anopheles funestus* s.s.	73	Ahero (73)	*Microsporidia MB* in 5 specimens
*Mesocyclops* sp.	34	Ahero (5) and Mwea (29)	No infection
*Macrocyclops sp.*	51	Ahero (20) and Mwea (31)	No infection
*Daphnia* sp.	20	Ahero (15) and Mwea (5)	No infection

## Discussion

The results clearly demonstrate that *Microsporidia MB* is transmitted horizontally between adult *An. arabiensis*. Transmission was only observed in cages that had opposite sexes of *Microsporidia MB* infected and uninfected adult *An. arabiensis* suggesting that *Microsporidia MB* is transmitted sexually. *Anopheles gambiae s.l.* males package seminal fluid that is produced in the male accessory glands into a coagulated mating plug that is digested in the female atrium several days after mating (Giglioli and Mason, 1966). Sperm received by mated female *Anopheles* are stored in a dedicated organ called the spermatheca, which is relied upon by *Anopheles gambiae s.l.* females for a lifetime of offspring production (Tripet et al., 2003). We observed that *Microsporidia MB* intensity was much higher in the midgut and male gonads than in the carcass of male *An. arabiensis*. This suggests that *Microsporidia MB* either migrates to or proliferates in the male gonad. We observed multinucleate Microsporidia cells only in specimens that were infected with *Microsporidia MB*, indicating that these cells are developmental stages of *Microsporidia MB*. *Microsporidia MB* cells were specifically localized to the *An. arabiensis* male ejaculatory duct. The *Microsporidia MB* cells observed had either 4 or 8 nuclei, which likely indicates that *Microsporidia MB* sporogenesis is occurring in the *An. arabiensis* male ejaculatory duct as 4-nuclei sporogonial plasmodia develop into an 8-nuclei stage and finally to become sporophorous vesicles. This developmental sequence has been reported in greater detail in Microsporidians associated with fire ants (Sokolova and Fuxa, 2008) and Daphnia (Refardt et al., 2008). It is therefore likely that the sporogenesis of *Microsporidia MB* in the male ejaculatory duct produces infectious spores that are released with seminal secretions and therefore transferred to females upon mating. Transmission from female to male *An. arabiensis* was also observed, but further investigation will be required to establish the basis of this transmission route.

Two findings indicate that mating is required for *Microsporidia MB* transmission. Firstly, the absence of *Microsporidia MB* transmission in same sex transmission cage experiments, and secondly the finding that in the three cage experiments where female *An. arabiensis* spermatheca were checked for the presence of sperm, only inseminated females acquired *Microsporidia MB*. The experimental design precluded the quantification of precise transmission rates, since in the majority of experiments female insemination events were not confirmed. However, in light of the low rate of female insemination in cages where spermatheca were checked, it can be expected that the rate of *Microsporidia MB* transmission from males to female *An. arabiensis* per successful mating is likely to be high.

The number of females infected and the intensity of *Microsporidia MB* infections in recipient females was not dependent on the intensity of *Microsporidia MB* in donor males. A possible explanation for this finding is that *Microsporidia MB* are localized to midguts and gonads. It is possible that localization to the male gonad is a pre-requisite for sexual transmission and that only the intensity of *Microsporidia MB* infection in gonads is correlated with transmission capacity. The finding that *Microsporidia MB* intensity was high in the midgut and that some male *An. arabiensis* had high intensity of *Microsporidia MB* only in the midgut suggests that this organ may play a yet to be determined role in transmission or alternatively that the midgut is a reservoir of *Microsporidia MB.* Notably, since the majority of gonadal tissue development occurs during metamorphosis, localization to the midgut could be required for maintenance of *Microsporidia MB* infection in *An. arabiensis* larval stages.

From the perspective of symbionts that are strictly maternally inherited, males are a dead end. In many cases, including for maternally inherited microsporidians, this can lead to the evolution of feminization or male-killing (Ironside and Alexander, 2015). Another possible outcome is that maternally inherited infections evolve to become sexually transmitted. The sexual transmission of beneficial heritable microbes has been reported in aphids (Moran and Dunbar, 2006). It is probable that in aphids sexual transmission enabled decreased pathogenicity of symbionts and co-evolution toward obligate mutualism.

Sexual horizontal transmission has been reported in a variety of insect-associated microsporidians (Knell and Mary Webberley, 2004) and in most cases it is associated with other complementary forms of transmission. Sexual transmission is likely to be more effective in insect species that have overlapping generations and higher levels of promiscuity. In *Anopheles*, the bacterial symbionts *Asaia* (Favia et al., 2007) and *Serratia* AS1 (Wang et al., 2017) have been shown to be sexually transmitted. It is notable that *Anopheles gambiae s.l.* is largely monandrous and therefore it is unlikely that symbionts could rely solely on sexual horizontal transmission. Indeed, both *Asaia* and *Serratia* AS1 are also transmitted vertically and by other horizontal transmission routes. It is notable that sexually transmitted infections of insects tend to reach much higher prevalence levels than infections with other forms of horizontal transmission (Knell and Mary Webberley, 2004). An example is the Microsporidian *Nosema calcarati*, which is sexually and vertically transmitted in its host *Pitogenes calcaratus* and found at a prevalence of 50% (Purrini and Halperin, 1982). High prevalence may be in part due to the fact that sexual transmission selects for lower levels of virulence toward the hosts. Sexually transmitted infections can manipulate insect host physiology or behavior to favor higher levels of transmission, for example sexually transmitted mites were shown to increase the mating success of male midge hosts (McLachlan, 1999). Whether any sexually transmitted pathogens of *Anopheles* affect mating behavior has not been established.

*Microsporidia MB* was not found in non-anopheline arthropods that are found in the same habitats as *An. arabiensis* larvae. Since *Microsporidia MB* is transmitted vertically and by sexual horizontal transmission, a high level of host specificity could be expected since neither vertical (Herren et al., 2020), nor sexual horizontal transmission would be effective across species. It is noteworthy that *Microsporidia MB* was found in another species of anopheline mosquito, *An. funestus s.s.*, which is a primary vector of malaria in Sub-Saharan Africa. If the *Microsporidia MB* found in *An. funestus s.s* have similar characteristics to *Microsporidia MB* found in *An. arabiensis*, including *Plasmodium* transmission blocking, then *Microsporidia MB* could be developed as a tool for malaria control in several primary vector species.

To be successfully developed into a strategy to control malaria, an effective method of disseminating *Microsporidia MB* into *Anopheles* populations will need to be established. Our results show that *Microsporidia MB* infected male mosquitoes can infect their female counterparts and that horizontally infected females can transmit *Microsporidia MB* to their offspring. We previously showed that *Microsporidia MB* is vertically transmitted in *An. arabiensis* (Herren et al., 2020) and therefore *Microsporidia MB* infected males for releases could be produced by sorting the offspring of *Microsporidia MB* infected *An. arabiensis* colonies. These findings could be the basis for a dissemination strategy that involves targeted release of *Microsporidia MB* infected male *Anopheles* mosquitoes, potentially avoiding the need to release biting females, which would be advantageous in terms of community engagement and acceptance of the intervention. In principle, such a strategy would be similar to the mass-release of sterile males (Bouyer et al., 2020), except that instead of sterilizing females *Microsporidia MB* infected males would decrease the capacity of infected females and their offspring to transmit malaria for multiple generations. The capacity of *Microsporidia MB* to be vertically transmitted after infecting females would potentially make this approach more sustainable and cost-effective than SIT.

## Materials and Methods

### Field Collections

Resting gravid and engorged female mosquitoes were collected indoors through manual aspiration. Collections were undertaken in Ahero (–34.9190W, –0.1661N) and Mwea (–37.3538W, –0.6577N) between Feb and June 2020 between 0630 h and 0930 h using electric torches/lights and aspirators. Collected females were placed in large cages supplied with 6% glucose and transported to *icipe*-Thomas Odhiambo Campus (*iTOC*) from Ahero and *icipe* Duduville campus from Mwea for processing. Mosquito larvae and other organisms were collected from larval habitats in Mwea and Ahero using larval collection dippers between March and July 2019. *Anopheles funestus* and *coustanii* were collected in November 2018 in Ahero as adults in houses (for *Anopheles funestus*) and cattle-baited traps (for *Anopheles coustanii). Aedes aegypti* and *Culex* sp. larval stages were collected in March 2018 from old discarded wheel tires in Kilifi and Malindi and transported to the rearing facility at Pwani University for emergence.

### Mosquito Identification, Processing, and Rearing

All transmission experiments were carried out on wild-collected *Anopheles gambiae sl*., which were identified morphologically. In all of the collection sites, *An. arabiensis* is the most common member of the *An. gambiae* species complex, with >97% of complex members being identified as *An. arabiensis*. The high percentage of *An. arabiensis* in field collections from both sites was re-confirmed using PCR (Santolamazza et al., 2008). *An. funestus* species were identified by PCR (Koekemoer et al., 2002). Wild collected mosquitoes were maintained in an insectary at 27 ± 2.5^o^C, humidity 60–80% and 12-h day and 12-h night cycles and induced to oviposit in individual microcentrifuge tubes containing a wet 1 cm × 1 cm Whatman filter paper. Eggs from each female were counted under a compound microscope using a paint brush and then dispensed into water tubs for larval development at 30.5°C and 30–40% humidity. Tetramin^TM^ baby fish food was used to feed developing larvae. Upon laying eggs, the G_0_ females were screened for presence of *Microsporidia MB* by PCR. The larval offspring of *Microsporidia MB* positive field-caught female mosquitoes were pooled into larval rearing troughs for experimentation. *Microsporidia MB* uninfected controls were obtained from the *An. arabiensis* colonies at *icipe iTOC* Mbita and Duduville campuses.

### Inoculation of *Microsporidia MB* Homogenate by Feeding

Five infected *An. arabiensis* larvae or adults were placed in 1.5 ml microcentrifuge tubes containing 500 μl 1 × PBS. *An. arabiensis* larvae and adults were homogenized using a pestle and then transferred directly into larval rearing water or sugar sources. For larval rearing water, 500 μl homogenate was added to 500 ml of distilled water at the L2 larval stage. For adults, 250 μl homogenate was added to 20 ml of 10% sucrose solution. Recipient larvae that developed in rearing water with homogenate were screened as 1–2 day old adults. Recipient adults were screened 2 days after initial homogenate exposure. Aliquots of the homogenate were kept at −20°C and screened by PCR, all homogenates used were *Microsporidia MB* positive.

### Transmission Between Live *An. arabiensis* Larvae

*Microsporidia MB* infected donor and uninfected recipient *An. arabiensis* larvae (donor *N* = 7–20 and recipient *N* = 9–31) were transferred into a 15 cm x 30 cm larval rearing trough that had a 70 μm mesh divider between two sections. Donor and recipient larvae were placed in separated sections and maintained until they emerged as adults. Both donor and recipient *An. arabiensis* were screened as 1–2 day old adults to determine the percentage of donors that were infected and if recipients had horizontally acquired *Microsporidia MB*.

### Transmission Between Live *An. arabiensis* Adults

*Microsporidia MB* infected and uninfected *An. arabiensis* virgin adults were transferred into 30 cm × 30 cm × 30 cm cages. Virgin mosquitoes were obtained by separating the sexes at the pupal stage after visual examination of the terminalia. To increase the chances of observing transmission, several (2–6) *Microsporidia MB* infected *An. arabiensis* donors were kept with 12–25 virgin uninfected recipient mosquitoes for 2 days. In the experiments where sex could not be used to differentiate male and female mosquitoes, dyes (red and blue) were used to mark mosquito wings and indicate donors and recipients. Upon completion of the transmission experiment all *An. arabiensis* mosquitoes were screened to determine the percentage of donors that were infected and if recipients had horizontally acquired *Microsporidia MB*. To investigate the efficiency of horizontal transmission and the importance of *Microsporidia MB* intensity, additional cages with single *Microsporidia MB* infected donor males and 10–50 virgin *Microsporidia MB* uninfected recipient females were established and maintained for 2 days. In the single infected donor male cage experiments, post exposure, female recipients were allowed to feed on a human arm for 15 min at 19:00 h. Mosquitoes that fed were placed in individual micro centrifuge tubes with wet filter papers to induce oviposition Upon completion of the transmission experiment all *An. arabiensis* mosquitoes were screened by qPCR to determine the infection status and intensity of donor and recipient *An. arabiensis*. The offspring from *Microsporidia MB* infected recipient females from single male cage experiments were reared until they were 1–2 day old adults and then screened for *Microsporidia MB* to determine if vertical transmission had occurred. To investigate mating rates and the link between acquiring *Microsporidia MB* and female insemination status, the presence of sperm in *An. arabiensis* females maintained in some of the cages with *Microsporidia MB* infected males were examined by the dissection of spermathecae and scoring sperm presence.

### Quantification of *Microsporidia MB* Distribution Across Male *An. arabiensis* Organs

Quantification of *Microsporidia MB* was conducted on dissected organs from G_1_ *Microsporidia MB*-infected *An. arabiensis* adult males, 3–5 days post emergence. Midguts and gonads were separated from the remainder of the mosquito which was designated as the carcass. Each organ and the carcass was individually screened for *Microsporidia MB* presence and intensity by qPCR. Quantification of *Microsporidia MB* in the male seminal fluid was carried out on different *An. arabiensis* specimens. Briefly, 10–12 day old males were decapitated and used immediately in forced mating experiments with virgin females (full method given at www.mr4.org). Upon successful copulation, seminal secretions produced by the male were collected with a pulled capillary tube and transferred to a 10uL 1 × PBS and placed under ice. Genomic DNA was collected as previously described prior to *Microsporidia MB* quantification by qPCR.

### Microscopy of *An. arabiensis* Male Gonad

Microscopy was conducted on dissected G_1_ *Microsporidia MB*-infected and uninfected (control) *An. arabiensis* adult male gonads, 3–5 days post emergence. Gonads were fixed in 4% Paraformaldehyde (PFA) solution for 30 min. After three quick washes with PBS-T, samples were stained in 0.1mM Syto-9 in PBS for 1 h. After two quick washes and one 10 min wash, the gonads were placed on a slide and were visualized immediately using a Leica SP5 confocal microscope (Leica Microsystems, United States). Images were analyzed with the ImageJ 1.50i software package (Schneider et al., 2012).

### Specimen Storage and DNA Extraction

All *An. arabiensis* specimens were dry frozen at –20°C in individual microcentrifuge tubes prior to DNA extraction. DNA was extracted from each section individually using the protein precipitation method (Puregene, Qiagen, Netherlands).

### Molecular Detection of Presence and Intensity of *Microsporidia MB*

*Microsporidia MB* specific primers (MB18SF: CGCCGG CCGTGAAAAATTTA and MB18SR: CCTTGGACGTG GGAGCTATC) were used to detect *Microsporidia MB* in *An. arabiensis* larvae and adults (Herren et al., 2020). A 10 μl PCR reaction consisted of 2 μl HOTFirepol^®^ Blend Master mix Ready-To-Load (Solis Biodyne, Estonia, mix composition: 7.5 mM Magnesium chloride, 2 mM of each dNTPs, HOT FIREPol^®^ DNA polymerase), 0.5 μl of 5 pmol μl^–1^ of forward and reverse primers, 2 μl of the template and 5 μl nuclease-free PCR water was undertaken. Conditions used were initial denaturation at 95°C for 15 min, followed by 35 cycles of denaturation at 95°C for 1 min, annealing at 62°C for 90 s and extension at 72°C for a further 60 s. Final elongation was done at 72°C for 5 min. The intensity of *Microsporidia MB* infection was determined by a qPCR assay using MB18SF/MB18SR primers. These were normalized against the *Anopheles* ribosomal S7 host gene primers (S7F: ^5^′TCCTGGAGCTGGAGATGAAC^3^′ and S7R ^5^′GACGGGTCTGTACCTTCTGG^3^′, Dimopoulos et al., 1998).

### Statistical Analysis

We carried out statistical analyses using the two-tailed paired spearman’s rank test to compare paired donor and recipient *Microsporidia MB* intensity data values which had a non-normal distribution. To analyze if donor *Microsporidia MB* intensity or number of available mates affected the odds of *Microsporidia MB* transmission, a logistic regression analysis was carried out. All statistical analyses were undertaken using GraphPad Prism version 6.0c software and R (version 3.5.3). *P*-values of ^∗^*p* < 0.05, ^∗∗^*p* < 0.01, ^∗∗∗^*p* < 0.001, and ^****^p < 0.0001 were deemed to be statistically significant.

## Data Availability Statement

All the datasets presented in this study can be found an online repository: https://doi.org/10.6084/m9.figshare.14846925.v2.

## Author Contributions

JH and GN conceived and designed the majority of the experiments. GN, TM, TB, and EEM performed the majority of the experiments. GN, EEM, TM, DM, and EO collected mosquitoes and screened them for symbionts. JH, SS, EM, EEM, LM, ET, JP, JB, GN, TB, TO, FO, and GM analyzed the data. JH, TO, and FO carried out the microscopy. JH and GN wrote the manuscript.

## Conflict of Interest

The authors declare that the research was conducted in the absence of any commercial or financial relationships that could be construed as a potential conflict of interest.

## Publisher’s Note

All claims expressed in this article are solely those of the authors and do not necessarily represent those of their affiliated organizations, or those of the publisher, the editors and the reviewers. Any product that may be evaluated in this article, or claim that may be made by its manufacturer, is not guaranteed or endorsed by the publisher.

## Supplementary Material

Supplementary Figure 1 The intensity of *Microsporidia MB* in recipient females is not correlated to donor male intensity, with a regression slope that does not significantly differ from zero (*P* = 0.34, *r* = 0.177, and *n* = 31).
