# Supplementary figures and images for: Horizontal Transmission of the Symbiont Microsporidia MB in Anopheles arabiensis
Source: Front Microbiol. 2021 Jul 28;12:647183. doi: 10.3389/fmicb.2021.647183 (PMC8355901; doi:10.3389/fmicb.2021.647183)

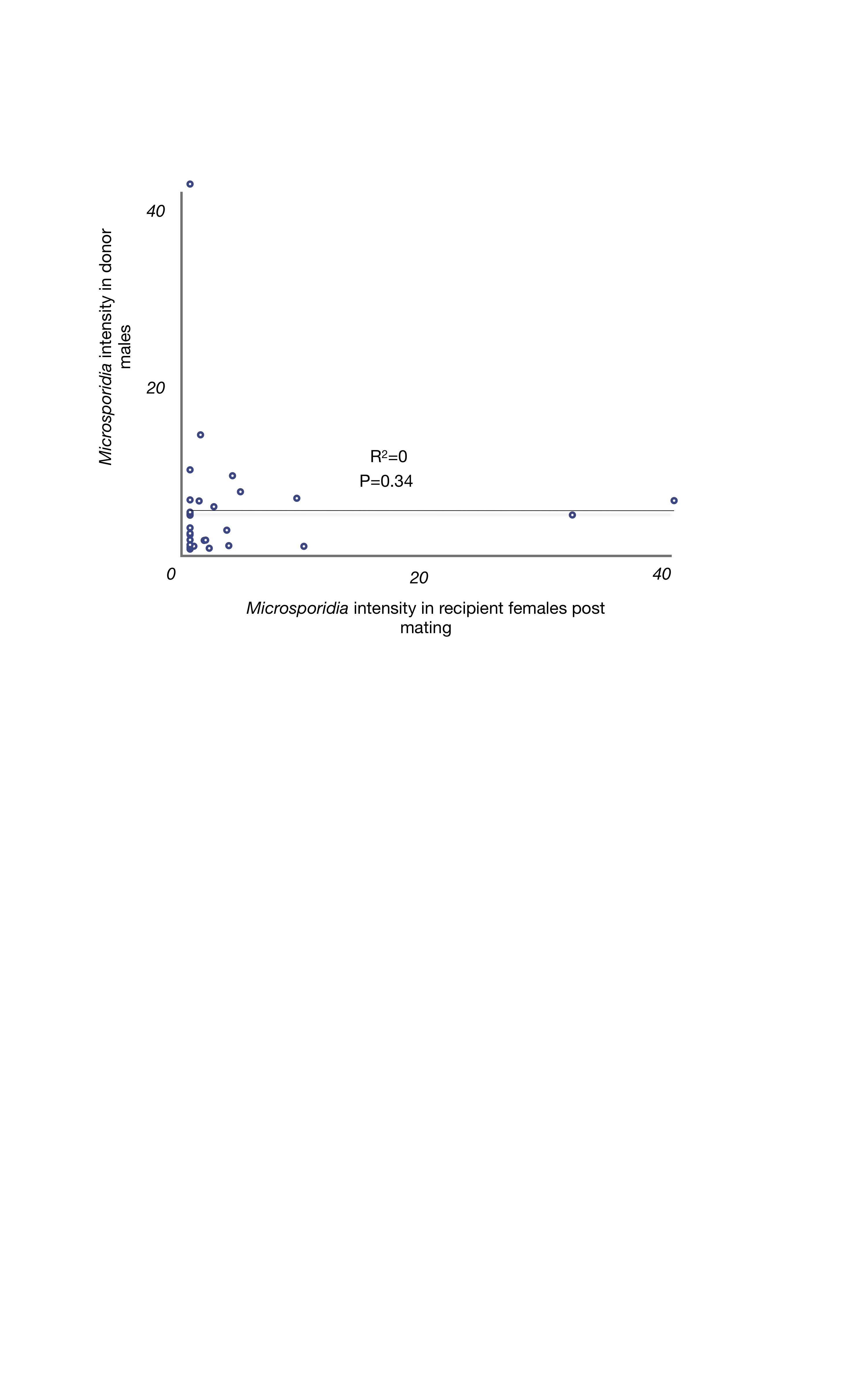

Supplement: Supplementary Figure 1 — The intensity of Microsporidia MB in recipient females is not correlated to donor male intensity, with a regression slope that does not significantly differ from zero (P = 0.34, r = 0.177, and n = 31). [file Image_1.JPEG]
